# The generative illusion: how ChatGPT-like AI tools could reinforce misinformation and mistrust in public health communication

**DOI:** 10.3389/fpubh.2025.1683498

**Published:** 2025-09-26

**Authors:** Jeena Joseph, Binny Jose, Jobin Jose

**Affiliations:** ^1^Department of Computer Applications, Marian College Kuttikkanam Autonomous, Kuttikkanam, Kerala, India; ^2^Department of Health and Wellness, Marian College Kuttikkanam Autonomous, Kuttikkanam, Kerala, India; ^3^Department of Library, Marian College Kuttikkanam Autonomous, Kuttikkanam, Kerala, India

**Keywords:** Generative AI, public health communication, misinformation, health literacy, low- and middle-income countries (LMICs), digital health equity

## Introduction

In the digital age, access to health information is no longer gated by clinics or libraries—it's increasingly mediated by algorithmic interfaces. The emergence of generative AI (GenAI) tools such as ChatGPT, Google Gemini, Claude, and open-source large language models has transformed how individuals seek, interpret, and act upon health information. Millions of users now consult AI models for everything from interpreting symptoms and planning diets to asking about medication side effects and mental health support (1).

While these tools promise democratization of knowledge and rapid access to information, their rise is not without peril—particularly in low- and middle-income countries (LMICs), where overburdened health systems, limited digital literacy, and high levels of medical misinformation already constitute a precarious information ecosystem (2). The illusion of authority presented by GenAI may inadvertently reinforce public health misinformation, deepen mistrust in formal medical systems, and introduce a new layer of algorithmic opacity in the way people manage health (3, 4).

This Opinion piece advocates an immediate focus on how generative AI tools, not even intended to be used in medicine, already pose significant challenges in ensuring accuracy, trust, and verification within public health communication. We cannot be content to correct biases coded into data and models; we also need to comprehend how tools get adopted, trusted, and responded to in actual world settings—least of all, by vulnerable individuals.

## The rise of Generative AI as a public health actor

Generative AI models function by predicting the next word in a sequence, based on vast corpora of text scraped from the internet. They lack factual grounding, medical training, or a capacity for real-time validation (5). Yet, their linguistic fluency and conversational tone lend them an aura of competence and confidence—traits that easily mislead users into believing that AI-generated answers are factual, current, and safe (6, 7).

In LMICs, where patients may face long wait times, limited access to healthcare providers, or language barriers in traditional health systems, GenAI presents an attractive alternative. It is free, fast, and available in multiple languages—qualities that formal health systems often fail to offer (8). In effect, generative AI tools have begun to function as de facto triage systems, digital advisors, and even emotional support systems for users with limited alternatives (9).

The problem is that these models are not trained to provide public health guidance. They do not reason medically, understand contextual risks, or tailor advice based on socioeconomic constraints. That said, many LLMs, including ChatGPT and Gemini, are capable of producing accurate and helpful health-related information when provided with sufficient context and detail. The limitation often lies less in the model's potential capacity and more in the variability of real-world inputs, where users may not supply adequate contextual information. A model that suggests an MRI for a headache may be technically accurate, but financially or logistically impossible in a rural Indian village or a refugee camp (10). In such cases, the issue reflects the gap between generalized outputs and localized feasibility, rather than an inherent flaw in the model itself. Worse still, these systems often “hallucinate”—confidently generating false or fabricated information, with no means of indicating uncertainty or danger (11, 12).

## Misinformation by design: the epistemic limits of AI

The core issue lies in the epistemology of generative AI. These systems are not truth-seekers; they are statistical parrots, designed to mimic language patterns, not validate facts. Consequently, misinformation is not a bug—it is a feature, or at the very least, an unavoidable byproduct of the model's design (13).

Numerous real-world tests have shown that LLMs provide inconsistent answers to health queries, often depending on prompt phrasing, model temperature settings, or user interaction history (14). One day, ChatGPT might say “garlic can help reduce blood pressure” and on another, it might declare that “there is no medical evidence for garlic use in hypertension.” Both responses sound plausible; neither is reliably sourced. For lay users, especially those with limited health literacy, these contradictions are not signals of unreliability but rather points of confusion—making them more susceptible to confirmation bias and less inclined to seek clarification from medical professionals (11, 15).

Furthermore, GenAI tools do not inherently warn users about the limitations of their output. While OpenAI, Google, and others now attach disclaimers or watermarks, these are easy to ignore and often poorly understood. Without contextualization, users may act on AI advice without realizing its provisional, non-clinical nature (16). This is particularly dangerous in regions where traditional sources of health misinformation—folk remedies, WhatsApp forwards, unverified YouTube channels—already saturate the information ecosystem.

## The trust trap: why GenAI feels more reliable than it is

A key reason GenAI is dangerous in public health communication is the trust trap it creates. Users tend to trust outputs that are coherent, confident, and delivered in personalized tones. This psychological effect, known as the authority bias, is compounded in digital environments where users cannot see the source, verify credentials, or cross-check claims (17).

For example, a rural teenager struggling with acne may ask a chatbot for advice. If the model offers a confident—but inaccurate—response about using toothpaste or lemon juice, the user may follow through, inadvertently harming themselves (18). A pregnant woman with limited antenatal care may rely on a chatbot for diet recommendations, unaware that cultural and nutritional needs vary drastically by geography. The risks compound when such advice delays actual medical consultation or undermines trust in formal systems (19).

Additionally, LLMs are optimized to maintain conversation—not to challenge users' dangerous assumptions. If a user types “Is it true that vaccines cause autism?” a well-trained model might respond with a refutation—but it might just as easily present a “neutral” summary of both sides, inadvertently legitimizing falsehoods (20). In a polarized or misinformed media climate, such neutrality is not balance; it is complicity.

## Digital divides meet algorithmic illusions

The interaction between digital inequality and algorithmic illusion is particularly acute in LMIC contexts. Generative AI systems require not just literacy but digital fluency—users must know how to frame questions, assess answers, and navigate ambiguity. Yet millions of people in these regions are still first-generation digital users, accessing AI through low-bandwidth phones or via intermediary platforms like voice assistants—often in contexts where the tools themselves are primarily trained on English-language data and Western cultural frameworks (21).

In such settings, linguistic and cultural mismatches are common. GenAI systems trained primarily in Western biomedical texts struggle to interpret or respond to indigenous health concepts, traditional medicine, or even regional dialects (22). Moreover, while many LLMs can interpret common terms such as garmi, tap, or bukhar, they often struggle with cultural nuances, idiomatic expressions, or context-specific health concepts—leading to responses that may be technically correct yet misaligned with local understandings (23).

This creates a practical challenge under the guise of inclusion. While generative AI tools may appear universally accessible, their outputs must always be treated as provisional and verified by qualified health professionals. For people in LMICs, recognizing AI as a supportive tool rather than an authoritative source is essential to ensure safe and reliable use.

## Health professionals at the crossroads: aid or adversary?

Healthcare professionals now face a new challenge: how to respond to patients who come with AI-informed expectations or anxieties. Doctors report increasing instances of patients quoting ChatGPT or Google Bard in consultations—sometimes to double-check advice, sometimes to challenge it. This can either empower shared decision-making or undermine clinical judgment, depending on how the interaction unfolds (9).

Public health workers are similarly impacted. Community health workers (CHWs), who form an essential part of the healthcare workforce and have significantly contributed to better health outcomes globally, may in some regions face training or resource constraints. In such contexts, they might turn to GenAI for support or documentation. However, without proper vetting or localization, they too may risk absorbing and disseminating inaccurate content, especially in low-literacy environments (24).

The integration of GenAI into health systems is therefore not merely a technological issue—it is a relational and epistemological shift. It alters the trust contract between health seekers and providers, shifts the locus of authority, and introduces a new actor—algorithmic, faceless, unverifiable—into the most intimate realms of human vulnerability: illness, pain, and hope (25). At the same time, generative AI is already demonstrating benefits, such as improving efficiency in U.S. healthcare systems through assistance with documentation, patient communication, and workflow management, as well as supporting academic work in universities. These developments illustrate its potential to augment human expertise when carefully implemented. Nevertheless, risks remain: some healthcare providers may lean on generative AI as a faster alternative to consulting senior colleagues or specialists, which could introduce medical errors. Beyond patient safety, such shortcuts may reduce opportunities for collaborative clinical discussions, long a source of innovation and novel insights in medicine.

## Toward a framework of responsible AI use in public health

Given the urgency and ubiquity of GenAI adoption, public health systems must act swiftly to prevent harm. A coherent framework should include the following pillars:

- Digital Health Literacy Campaigns: Public education programs should teach users to interpret GenAI outputs critically, recognize model limitations, and cross-check advice with trusted sources. These campaigns must be culturally and linguistically tailored, delivered via schools, community centers, and health outreach programs.
- Regulatory Guardrails: Governments must define clear boundaries for AI use in health information. Disclaimers are not enough. Generative tools should be required to detect and flag health-related queries, trigger warnings, and redirect users to certified medical sources. LMICs should develop local standards rather than import those from high-income countries.
- Clinician-AI Mediation Tools: Instead of resisting AI, public health systems could create validated, clinician-augmented GenAI interfaces. These tools would allow providers to co-author responses, correct misinformation, and personalize outputs, bridging the gap between digital advice and clinical judgment. Such interfaces must be designed under the directive of healthcare providers and public health experts, not engineers alone, to ensure ethical and clinically sound outputs.
- Localization and Language Inclusion: GenAI tools must be fine-tuned to support underrepresented languages, cultural contexts, and traditional health knowledge systems. This requires open-access datasets, community partnerships, and inclusive AI governance mechanisms.
- Fact-Checking and Algorithm Auditing: Independent agencies should conduct regular audits of GenAI health outputs across multiple domains. A “nutrition label” for AI tools—indicating accuracy rates, known biases, and version history—can help demystify performance for public users. Another key step is ensuring that AI-generated information or advice includes references or citations. For example, the European Society of Cardiology has developed an AI chatbot for healthcare providers and researchers that cites the sources of its information, offering a model for transparency and accountability.

## Conclusion: rethinking trust in the age of machines

Generative AI will not disappear. If anything, it will become more sophisticated, persuasive, and embedded in the daily lives of millions. The challenge is not whether to use AI in public health—it is how to ensure it supports equity, accuracy, and trust, rather than undermines them. Public health communication has always required clarity, cultural humility, and credibility. GenAI risks replacing these with fluency, convenience, and charisma—qualities that are compelling but not always truthful. Unlike healthcare providers, who are explicitly bound by the Hippocratic Oath and established medical ethics frameworks, the engineers who design generative AI tools operate under different professional codes. This highlights the importance of ensuring that AI development and deployment adhere to ethical standards appropriate for health contexts, with patient safety and public wellbeing at the core. At the same time, caution is needed within the profession itself: some healthcare providers—particularly physicians—may be tempted to use generative AI as a quick substitute for consulting senior colleagues or specialists, which risks medical errors and undermines quality of care. Moreover, complex case discussions among healthcare providers have historically been a source of novel insights and innovations in medicine. Replacing such collaborative human interactions with human–AI exchanges may inadvertently hinder medical progress and innovation. In the hands of unprepared users, GenAI is not just a tool; it is a double-edged instrument capable of amplifying both access and alienation.

We must therefore approach generative AI not as a neutral innovation, but as a public health actor—one that interacts with complex human systems and socio-technical histories. To do so requires humility from developers, vigilance from policymakers, creativity from educators, and collaboration across disciplines. The stakes are too high for complacency. The illusion of help must not eclipse the reality of harm. If we wish to harness the generative future for good, we must invest in critical infrastructure, participatory design, and epistemic justice—so that the next frontier of public health is not just algorithmically advanced, but humanely aligned.
